# Supplementary material for: Signal Deconvolution and Noise Factor Analysis Based on a Combination of Time–Frequency Analysis and Probabilistic Sparse Matrix Factorization
Source: Int J Mol Sci. 2020 Apr 23;21(8):2978. doi: 10.3390/ijms21082978 (PMC7215856; doi:10.3390/ijms21082978)
Supplement: Supplementary file 1 [file ijms-21-02978-s001.pdf]

Supplementary material for

Signal Deconvolution and Noise Factor Analysis based on a Combination of Time–Frequency Analysis and Probabilistic Sparse Matrix Factorization

Shunji Yamada [1,2], Atsushi Kurotani [2], Eisuke Chikayama [2,3] and Jun Kikuchi [1,2,3,*]

[1] Graduate School of Bioagricultural Sciences, Nagoya University, Furo-cho, Chikusa-ku, Nagoya 464-8601, Japan; shunji.yamada@riken.jp (S.Y.)
[2] RIKEN Center for Sustainable Resource Science, 1-7-22 Suehiro-cho, Tsurumi-ku, Yokohama 230-0045, Japan; atsushi.kurotani@riken.jp (A.K.); chikaya@nuis.ac.jp (E.C.)
[3] Department of Information Systems, Niigata University of International and Information Studies, 3-1-1 Mizukino, Nishi-ku, Niigata 950-2292, Japan
[4] Graduate School of Medical Life Science, Yokohama City University, 1-7-29 Suehiro-cho, Tsurumi-ku, Yokohama 230-0045, Japan

* Correspondence: jun.kikuchi@riken.jp (J.K.); +81-45-508-9439 (J.K.)

Supplementary material

In this document, we provide the following information:

1. The mathematical theory of signal deconvolution
2. Supplementary figures and tables
3. References

An NMR measurement informatics tool is available at http://dmar.riken.jp/NMRinformatics/.

1. The Mathematical Theory of Signal Deconvolution

In this study, signal deconvolution was applied to free induction decays (FIDs) of one-dimensional (1D) nuclear magnetic resonance (NMR) to separate components and improve the signal-to-noise ratio (SNR). The mathematical theory underlying this signal deconvolution is based on the combined methods of short-time Fourier transform (STFT) and probabilistic sparse matrix factorization (PSMF).

In Fourier transform (FT) NMR spectroscopy, an FID is the NMR signal generated by non-equilibrium nuclear spin magnetization precessing along the magnetic field. This non-equilibrium magnetization can be generated by applying a pulse of resonant radiofrequency close to the Larmor frequency of the nuclear spins in the sample. An FID is usually a sum of multiple decayed oscillatory signals. These signals return to equilibrium at different rates or relaxation time constants. Analysis of the relaxation times of an FID for a sample gives significant insight into the chemical composition, structure, and mobility of that sample. FIDs acquired by NMR measurement are composed of many signals derived from the sample and several types of noise, such as external noise, physical vibration, power supply, and internal noise of the spectrometer due to thermal noise. Therefore, an FID signal can be modeled as:

$$S(t) = S_{signal}(t) + S_{noise}(t). \quad \text{(S1)}$$

where $S(t)$ is the measured signal, and $S_{\text{signal}}(t)$ and $S_{\text{noise}}(t)$ represent a set of ideal signals and a set of signals from different types of noise (Equation (S1)) [1]. Suppose that a 90° pulse is applied to an equilibrium magnetization along the *z*-axis, resulting in magnetization of the *x*–*y* plane, which then precesses in the transverse plane with angular frequency Ω. The corresponding time-domain signal that decays with time *t* is the FID *S*(*t*). In principle, the exponential decay constant of the FID is the T_2 relaxation time, which is a physically parameter independent of field inhomogeneity. In reality, however, because of the effect of magnetic field homogeneity, the decay constant of the FID is called T_2*, an instrument-dependent parameter, rather than T_2. *S*(*t*) is given by the relaxation time constant T_2^* [2]:

$$S(t) = S_0 \exp(i\Omega t) \exp\left(-\frac{t}{T_2^*}\right), \quad \text{(S2)}$$

where S_0 is the initial transverse magnetization at *t* = 0 immediately after the 90° pulse (Equation (S2)). The relaxation process can be described by saying that the transverse magnetization $S(t)$ decays exponentially according to Equation (S2). The shorter the relaxation time T_2^*, the more rapid the decay.

If an FID has more than one component, the FID will be the sum of contributions from each component (Equation (S3)):

$$S(t) = \sum_{k=1}^{n} S_{0k} \exp(i\Omega_k t) \exp\left(-\frac{t}{T_{2k}^*}\right). \quad \text{(S3)}$$

When there are two or more types of component (i.e., *k* > 1) in the FID signal, it is difficult to determine the individual signals from the time-domain signal $S(t)$. Therefore, we apply FT to $S(t)$ to yield a frequency-domain spectrum $S(\omega)$ with an angular frequency variable ω on the horizontal axis and *k* peaks at Ω_k (Equation (S4)):

$$S(\omega) \; = \; \int_{-\infty}^{\infty} S(t) \exp(-i\omega t) dt. \quad \text{(S4)}$$

Standard FT (Equation (S4)) has only has the frequency domain; therefore, we apply STFT, which has both frequency and time domains. Because the FID signal decays exponentially with time, for STFT, it needs to be divided into several small time intervals (i.e., segments) to analyze

the time–frequency feature accurately, and FT is used to determine the frequency feature of each segment, thereby increasing the accuracy of signal feature extraction. STFT uses a window function to obtain each weighted segment on the time axis and then applies FT to the segment. STFT of $S(t)$ can be written as:

$$STFT_S(\tau,\omega) = \int_{-\infty}^{\infty} S(t)g(t-\tau)\exp(-i\omega t)dt, \quad \text{(S5)}$$

where the window function g is first used to intercept the progress of FT on $S(t)$ around $t=\tau$ locally, and then FT of the segment is performed on t (Equation (S5)) [3]. By moving the center position of the window function g sequentially, all of the FTs at different times can be obtained.

Applying Euler's formula (Equation (S6)),

$$\exp(-i\omega t) = \cos\omega t - i\sin\omega t, \quad \text{(S6)}$$

shows that the value of $STFT_S(\tau,\omega)$ is complex and composed of two signals, a real part (Re) and an imaginary part (Im), whose phases differ by 90° from each other (Figure S1, Equation (S7) and (S8)):

$$Re = \gamma\cos\omega\tau, \quad \text{(S7)}$$

$$Im = \gamma\sin\omega\tau\,. \quad \text{(S8)}$$

To change a complex value into an absolute value, the following equation is applied (Equation (S9)):

$$|z| = \sqrt{Re^2 + Im^2}. \quad \text{(S9)}$$

For PSMF [4], positive-valued matrices are needed and the original signal values must be converted to their logarithmic form for optimal analysis. To convert Equation (S9) to a positive logarithmic form, the following equation is applied (Equation (S10)):

$$V = \log_{10}(|z| + 1). \quad \text{(S10)}$$

In our method using PSMF, we focus on sparse factorizations and on properly accounting for uncertainties while computing the factorization. Thus, signal deconvolution is formulated as finding the factorization of the data matrix V (Equation (S11)):

$$V = W \cdot H + residuals. \quad \text{(S11)}$$

When considering the separation of signal and noise, Equation (S11) can be described as the sum of a signal component, a noise component, and residuals (Equation (S12)):

$$V = W_{signal} \cdot H_{signal} + W_{noise} \cdot H_{noise} + residuals. \quad \text{(S12)}$$

Equation (S12) estimates that the signal component ($W_{signal} \cdot H_{signal}$) decays exponentially with time, while the noise component ($W_{noise} \cdot H_{noise}$) is a random or flat value. To reconstruct the FIDs, the absolute value within each component is converted back to a complex value using the following equations (Equation (S13) and (S14)):

$$Re = (10^{\log_{10}|z+1|} - 1)\cos\theta, \quad \text{(S13)}$$

$$Im = (10^{\log_{10}|z+1|} - 1)\sin\theta\,. \quad \text{(S14)}$$

The inverse short-time Fourier transform (ISTFT), $S_{inv}(t)$, is computed by overlap-adding the inverse fast Fourier transform signals in each segment of the STFT spectrogram as follows (Equation (S15)) [5]:

$$S_{inv}(t) = \int_{-\infty}^{\infty} \sum_{m=-\infty}^{\infty} V(\omega)\exp(i\omega t)d\omega. \quad \text{(S15)}$$

To evaluate SNR, both noise-removed and noise-only FIDs are converted to signal and noise spectra, respectively, by applying standard FT. SNR is calculated as the ratio of the signal peak intensity to the noise value by using the method of Mnova (Equation (S16)) [6]:

$$SNR = \frac{Signal\ peak\ intensity}{Noise\ value}. \tag{S16}$$

The noise value is calculated by using the standard deviation of the signals-free region (Equation (S17)):

$$Noise\ value = \sqrt{\frac{\sum_{i=1}^{N}(S(t)_i - S(t)_m)^2}{N-1}}, \tag{S17}$$

where N is number of points in the signal-free region, $S(t)_i$ is the value of each digital point in that region, and $S(t)_m$ is average of the digital points in that region.
Finally, the relative SNR is the ratio of the SNR after denoising ($SNR_{denoised}$) to the original SNR ($SNR_{original}$), which is calculated as follows (Equation (S18)):

$$Relative\ SNR = \frac{SNR_{denoised}}{SNR_{original}}. \tag{S18}$$

In order to obtain a theoretical SNR index based on acquisition parameters, the theoretical SNR value (*calcSNR*) was calculated by using a previously described formula (Equation (S19)) [7]:

$$calcSNR = \frac{C\gamma_{exc}T_2(\gamma_{det}B)^{3/2}\sqrt{NS}}{TE} \propto \frac{C(B)^{3/2}\sqrt{NS}}{TE\nu_{1/2}}. \tag{S19}$$

where, *C* is the number of spins in the system (sample concentration/number of protons), γ_{exc} is the gyromagnetic ratio of the excited nucleus, γ_{det} is the gyromagnetic ratio of the detected nucleus, *NS* is the number of scans, *B* is the external magnetic field, T_2 is the transverse relaxation time (the reciprocal of π times the line width at half height), *TE* is the sample temperature, and $\nu_{1/2}$ is the full width at half maximum.

2. Supplementary figures and tables

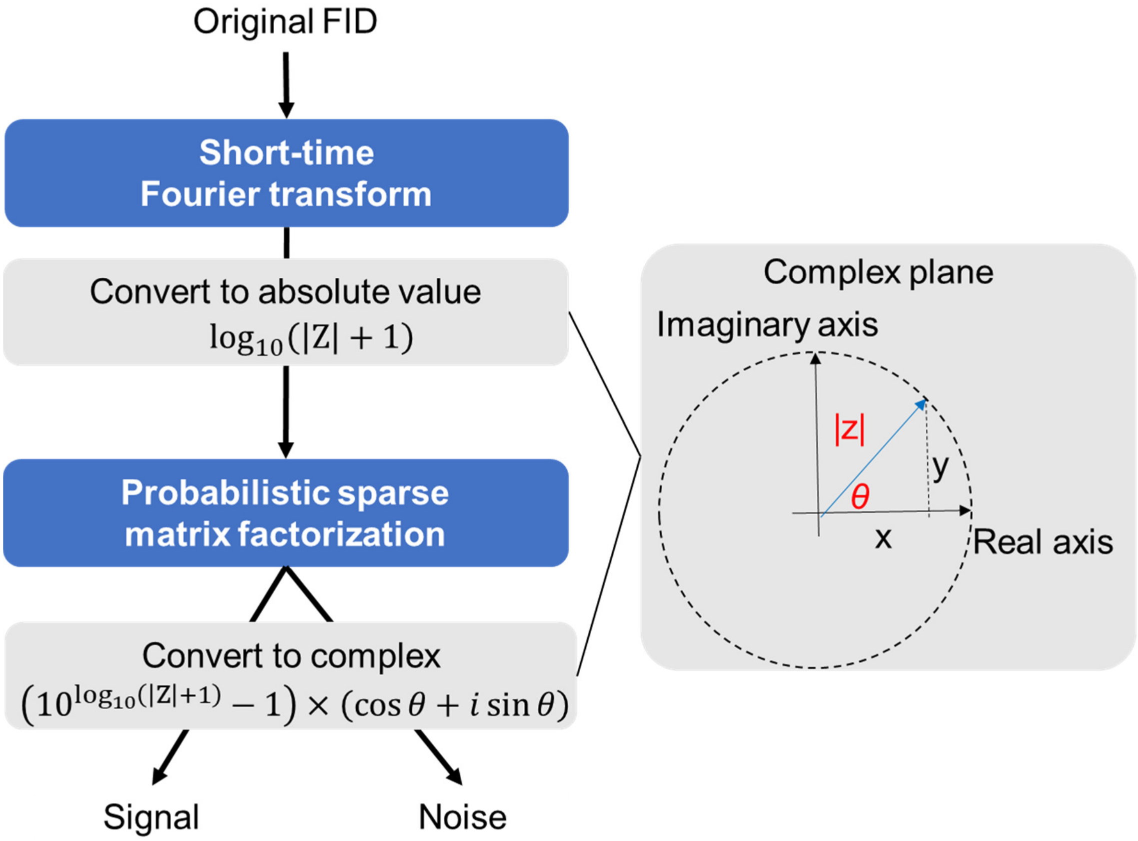

Figure S1: Schematic diagram showing the steps in the signal deconvolution method, including absolute value conversion and complex value conversion of the matrix. The original FID is subjected to STFT. The matrix of STFT is converted to an absolute value. This nonnegative value is separated to components of signal and noise by PMSF. The separated components are then converted to a complex value, from which denoised FIDs and time-domain noise data are extracted. The right image shows the relationship among the real part, imaginary part, absolute value, and argument in the complex plane.

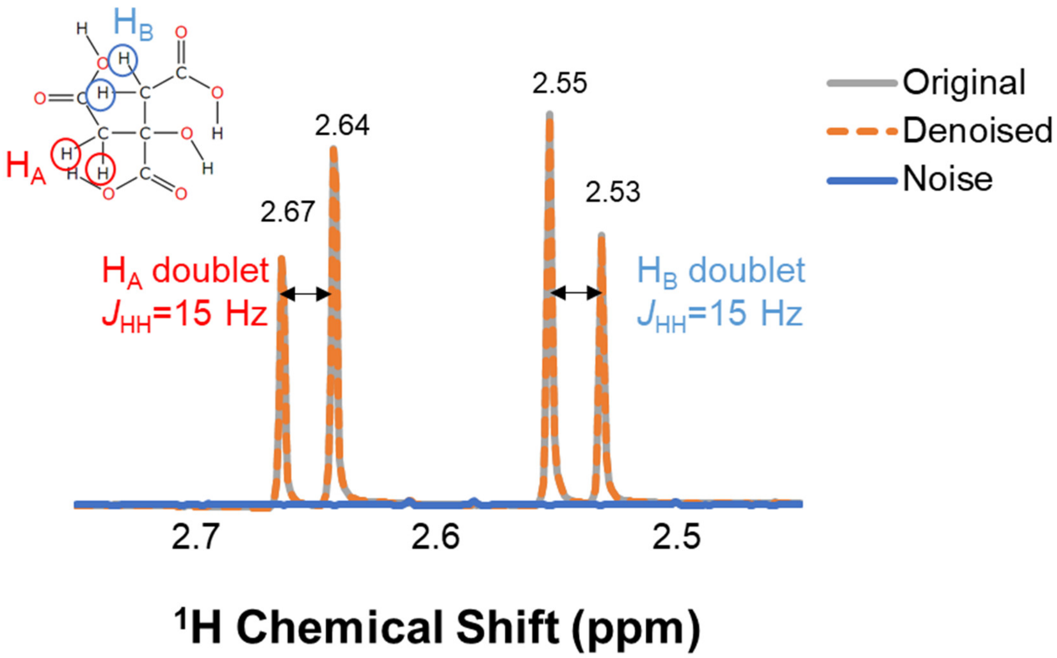

Figure S2: Original spectra and denoised spectra in ^{1}H-NMR data of citric acid. To demonstrate the denoising method, data for citric acid were acquired by using the presaturation (program name; "zgpr ") pulse sequence. The original spectrum (grey, solid line), denoised spectrum (orange, dashed line) and noise (blue, solid line) are shown. The chemical structure, peaks and *J* value of citric acid are shown in the figure. Information on the spectral values is shown in Table S1. Relative SNR of this spectra is 1.14-fold.

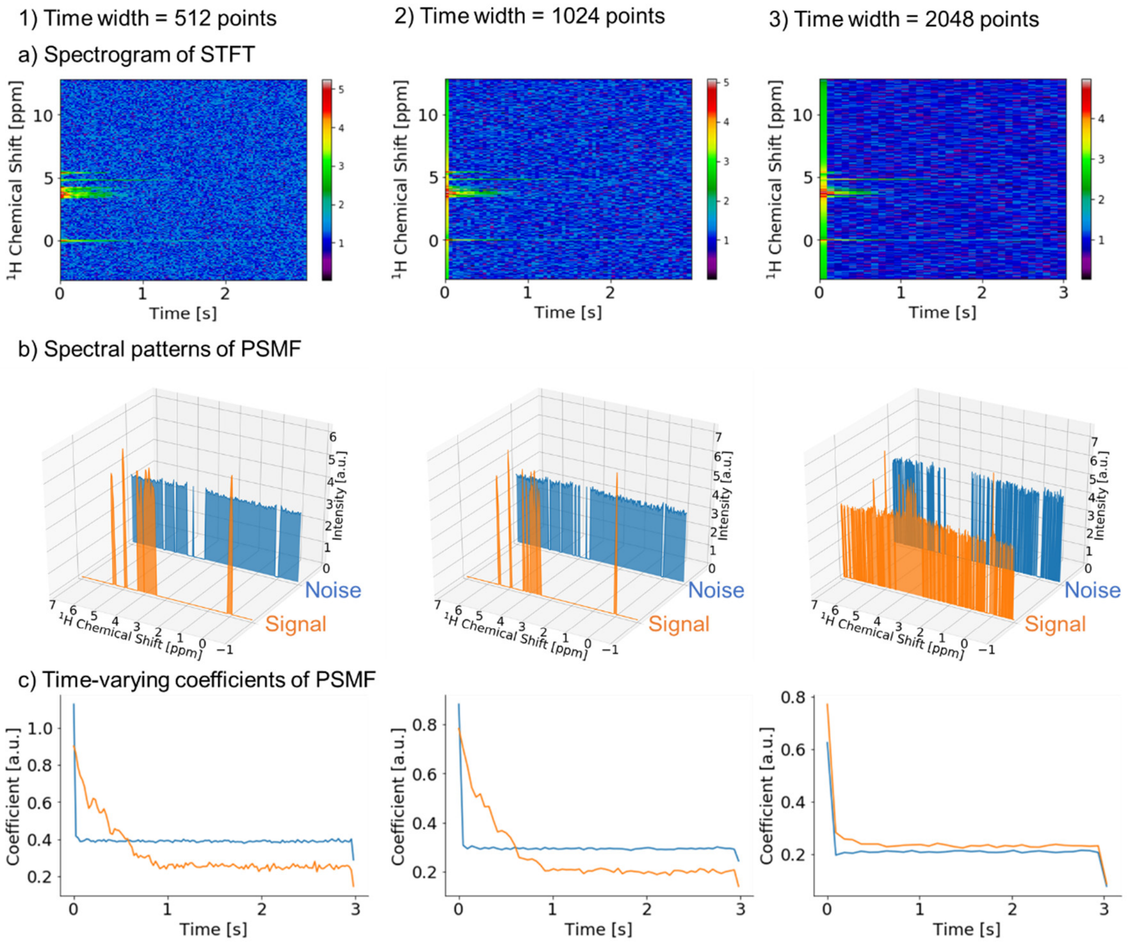

Figure S3: Effect of STFT time width on PSMF. STFT was performed using three different time widths, 512 points (1), 1024 points (2), and 2048 points (3), and the effect on separated components was investigated. a) Spectrogram obtained by STFT. b) Spectral patterns of PSMF. c) Time-varying coefficient of each component in PSMF.

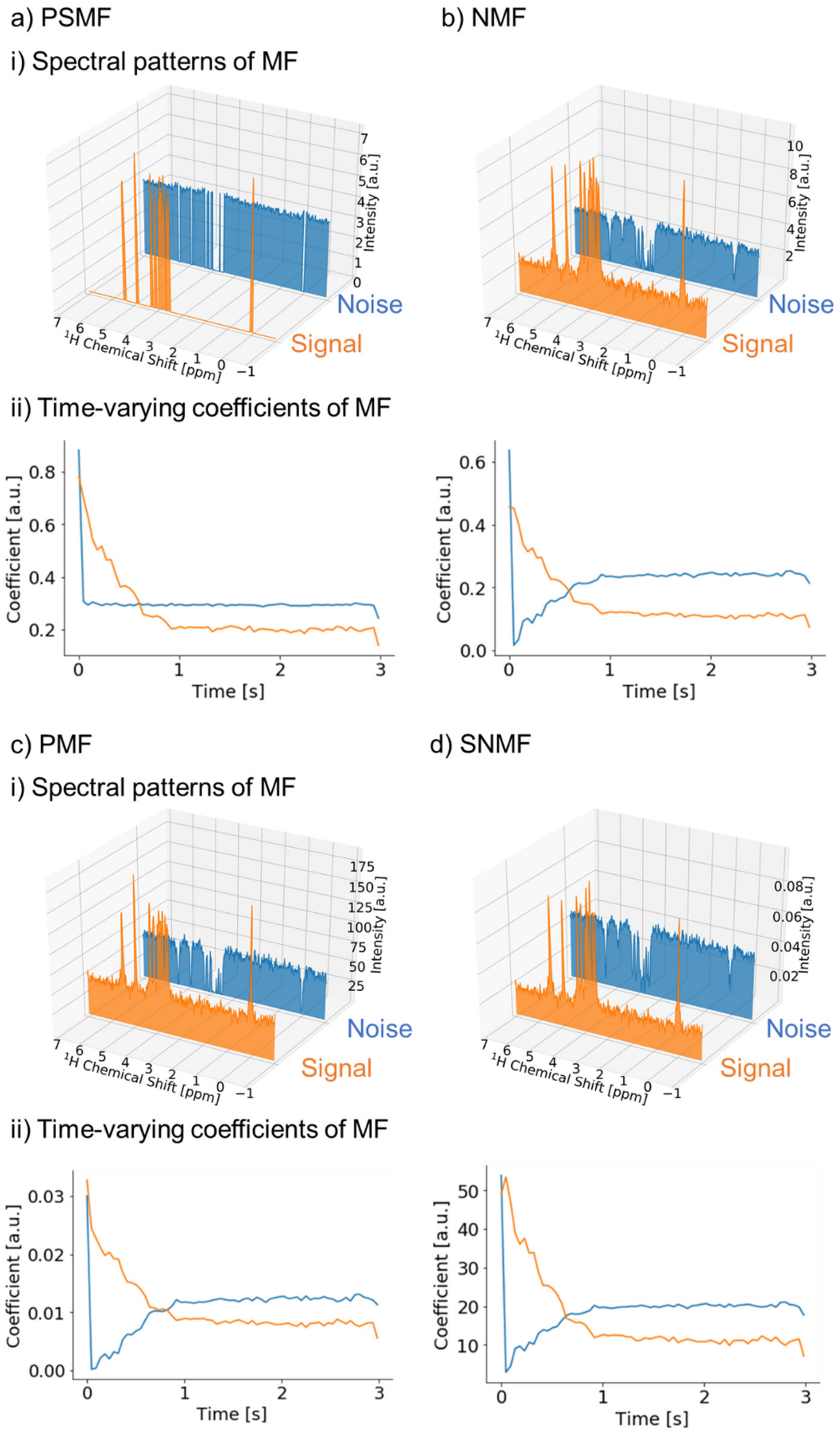

Figure S4: Comparison of four types of matrix factorization (MF) for signal deconvolution. MF was performed using four different methods, PSMF (1), NMF (2), PMF (3), and SNMF (4), and the effect on separated components was investigated. a) Spectral patterns of each MF method. b) Time-varying coefficient of each component in each MF method.

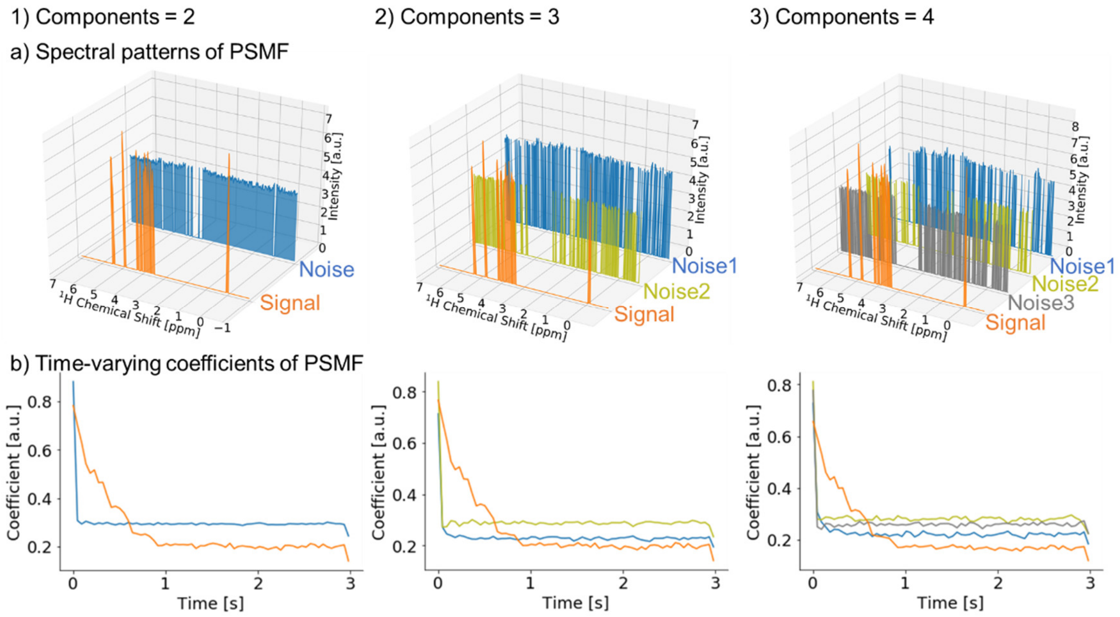

Figure S5: Effect of the number of components in PSMF. PSMF was performed using different numbers of components, two components (1), three components (2), and four components (3), and the effect on separated components was investigated. a) Spectral patterns of PSMF. b) Time-varying coefficient of each component in PSMF.

a) Heatmap of NMR data using CPMG

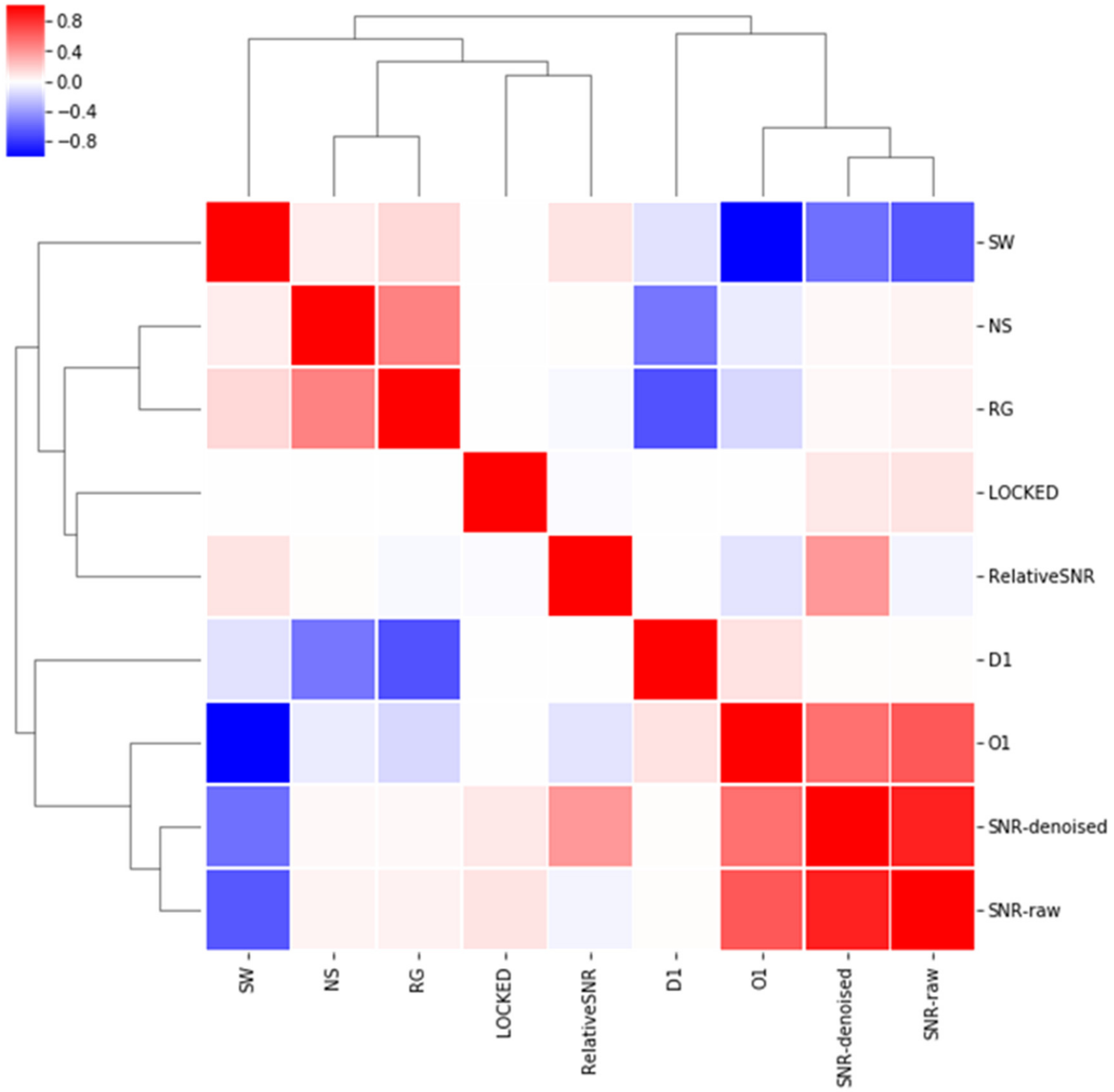

Figure S6a: Relationship between SNR and acquisition parameters of NMR data using CPMG. a) Heatmap. In the network diagram, positive correlations are red; negative correlations are blue; and the magnitude of the correlation coefficient is indicated by edge thickness. Abbreviations: SNR-raw, SNR of raw data; SNR-denoised, SNR of denoised data; RelativeSNR, relative SNR; RG, receiver gain; NS, number of scans; D1, relaxation delay time; SW, spectral width; O1, the offset of the transmitter frequency; LOCKED, if LOCK is on, value is 1, if not, value is 0.

b) Network diagram of NMR data using CPMG

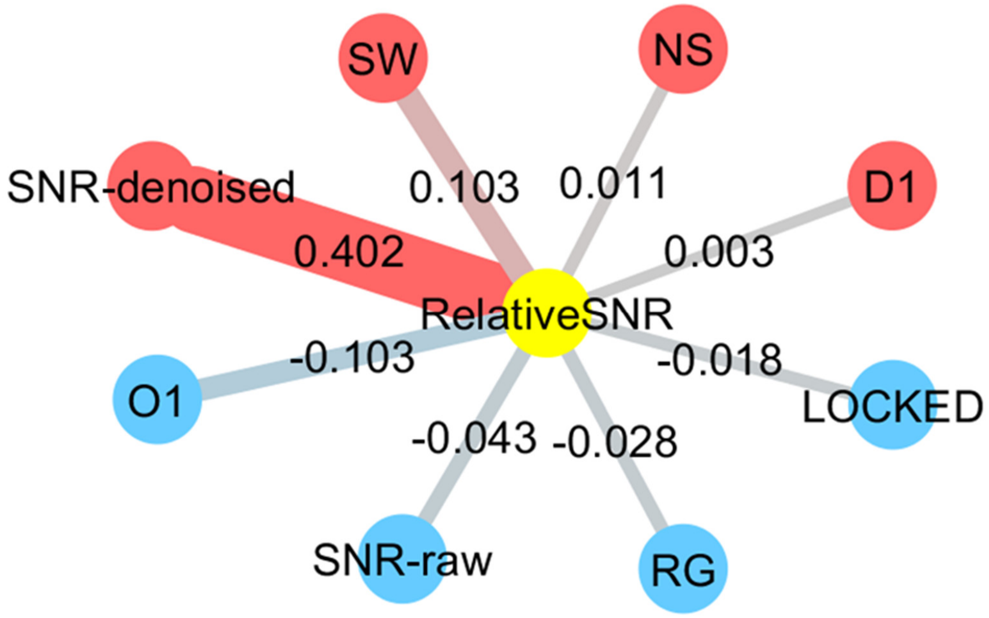

Figure S6b: Relationship between SNR and acquisition parameters of NMR data using CPMG. b) Network diagram. In the network diagram, positive correlations are red; negative correlations are blue; and the magnitude of the correlation coefficient is indicated by edge thickness. Abbreviations: SNR-raw, SNR of raw data; SNR-denoised, SNR of denoised data; RelativeSNR, relative SNR; RG, receiver gain; NS, number of scans; D1, relaxation delay time; SW, spectral width; O1, the offset of the transmitter frequency; LOCKED, if LOCK is on, value is 1, if not, value is 0.

a) Heatmap of NMR data using WATERGATE

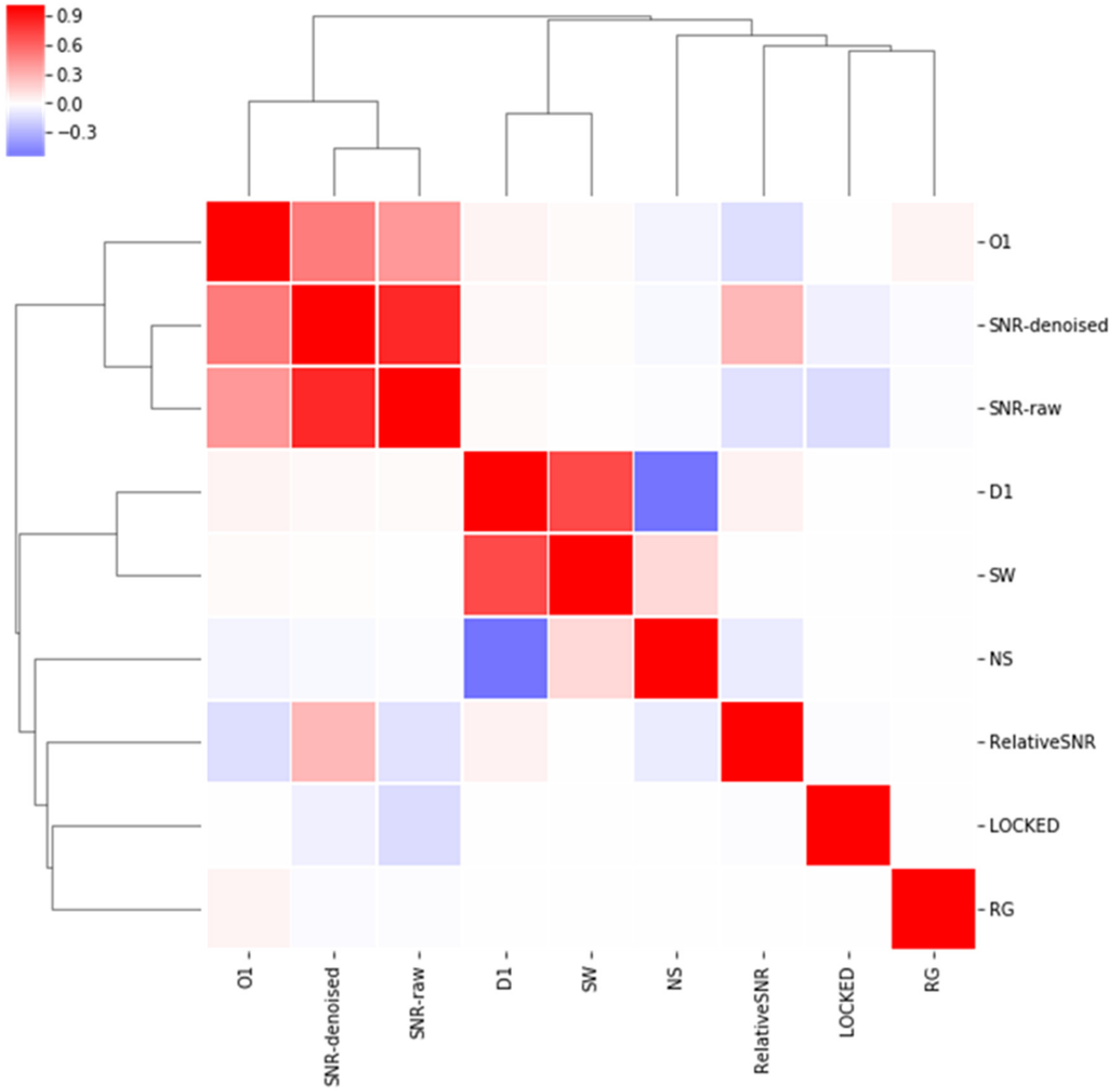

Figure S7a: Relationship between SNR and acquisition parameters of NMR data using WATERGATE. a) Heatmap. In the network diagram, positive correlations are red; negative correlations are blue; and the magnitude of the correlation coefficient is indicated by edge thickness. Abbreviations: SNR-raw, SNR of raw data; SNR-denoised, SNR of denoised data; RelativeSNR, relative SNR; RG, receiver gain; NS, number of scans; D1, relaxation delay time; SW, spectral width; O1, the offset of the transmitter frequency; LOCKED, if LOCK is on, value is 1, if not, value is 0.

b) Network diagram of NMR data using WATERGATE

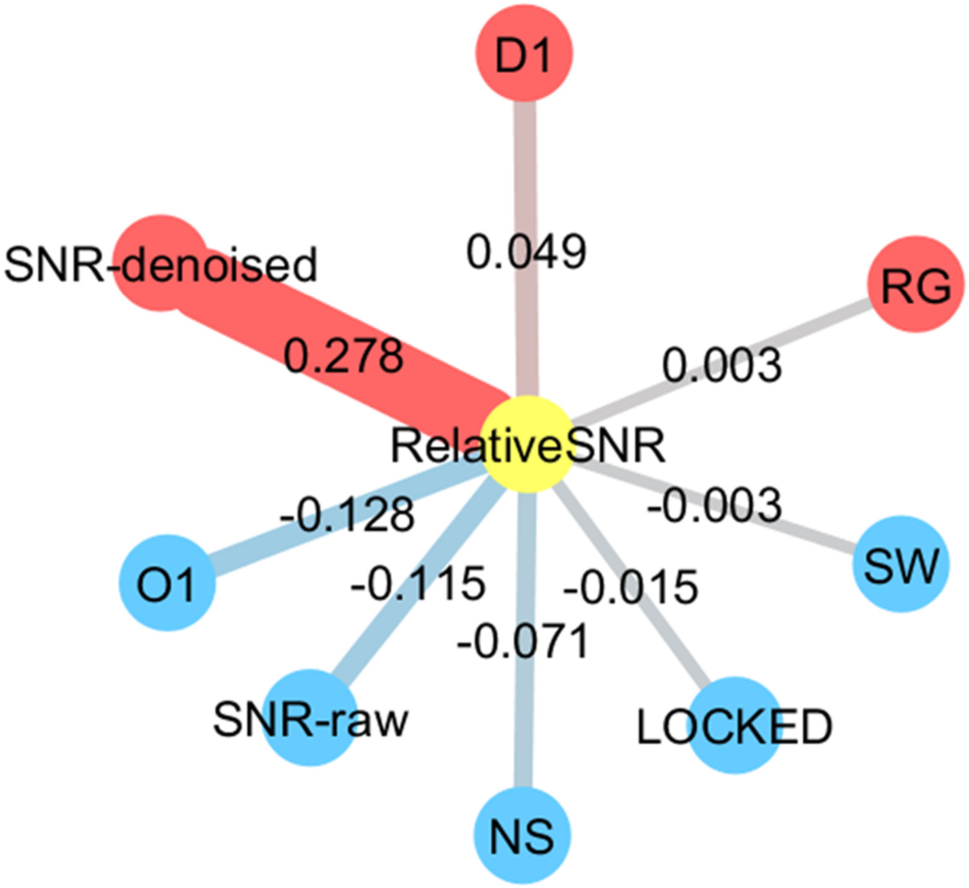

Figure S7b: Relationship between SNR and acquisition parameters of NMR data using WATERGATE. b) Network diagram. In the network diagram, positive correlations are red; negative correlations are blue; and the magnitude of the correlation coefficient is indicated by edge thickness. Abbreviations: SNR-raw, SNR of raw data; SNR-denoised, SNR of denoised data; RelativeSNR, relative SNR; RG, receiver gain; NS, number of scans; D1, relaxation delay time; SW, spectral width; O1, the offset of the transmitter frequency; LOCKED, if LOCK is on, value is 1, if not, value is 0.

a) Heatmap of diffusion-edited NMR

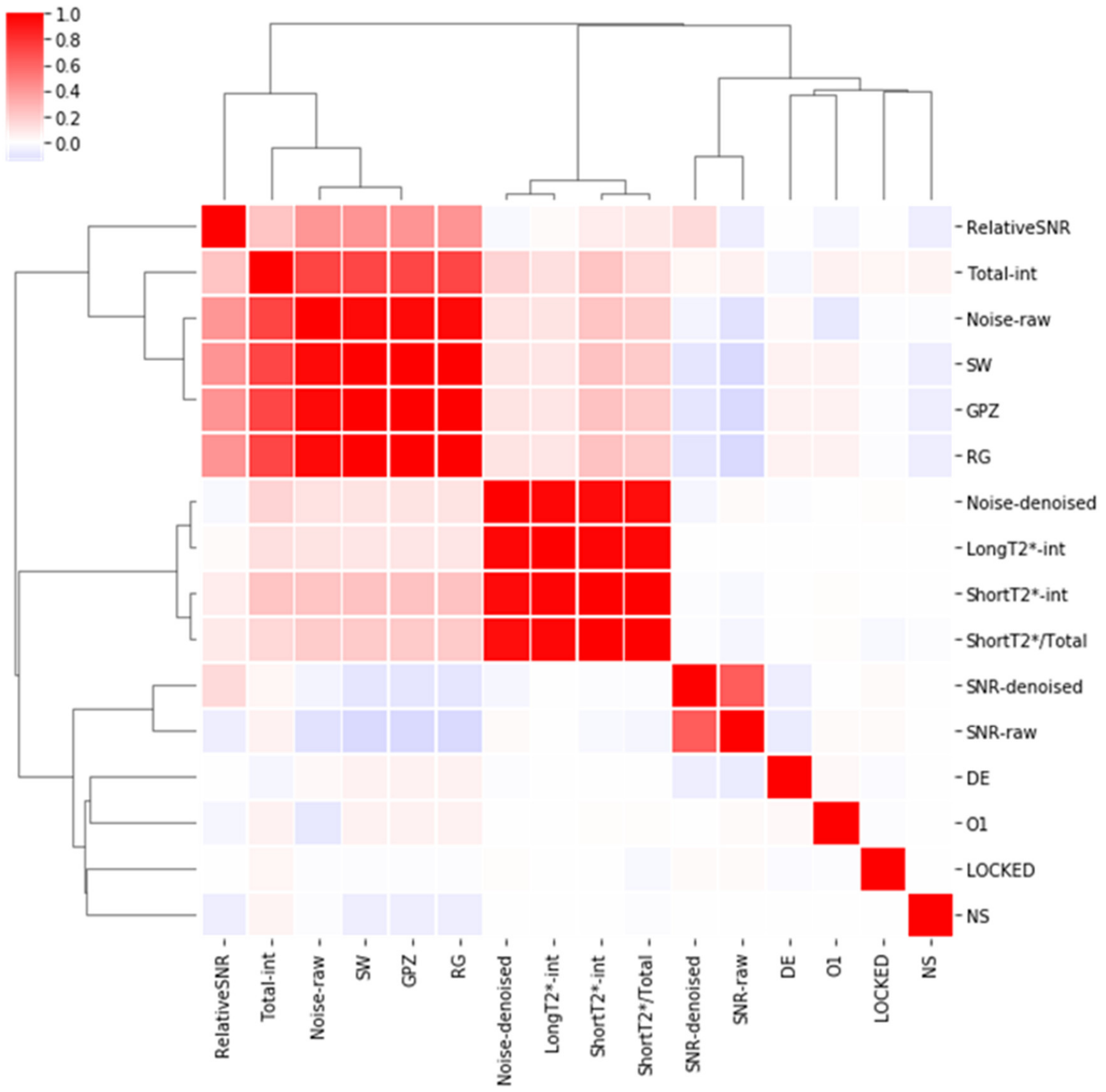

Figure S8a: Relationship between the data quality (SNR and the composition of the separated signal) and acquisition parameters of diffusion-edited NMR. a) Heatmap. In the network diagram, positive correlations are red; negative correlations are blue; and the magnitude of the correlation coefficient is indicated by edge thickness. Abbreviations: SNR-raw, SNR of raw data; SNR-denoised, SNR of denoised data; RelativeSNR, relative SNR; Total-int, total intensity; ShortT2*-int, intensity of short T_2^* signal; LongT2*-int, intensity of long T_2^* signal; ShortT2*/Total, ratio of intensity of long T_2^* signal to total intensity; Noise-raw, noise of raw data; Noise-denoised, noise of denoised data; GPZ, gradient pulse in the z-axis; RG, receiver gain; NS, number of scans; DE, pre-scan delay; SW, spectral width; O1, the offset of the transmitter frequency ; LOCKED, if LOCK is on, value is 1, if not, value is 0.

b) Network diagram of diffusion-edited NMR

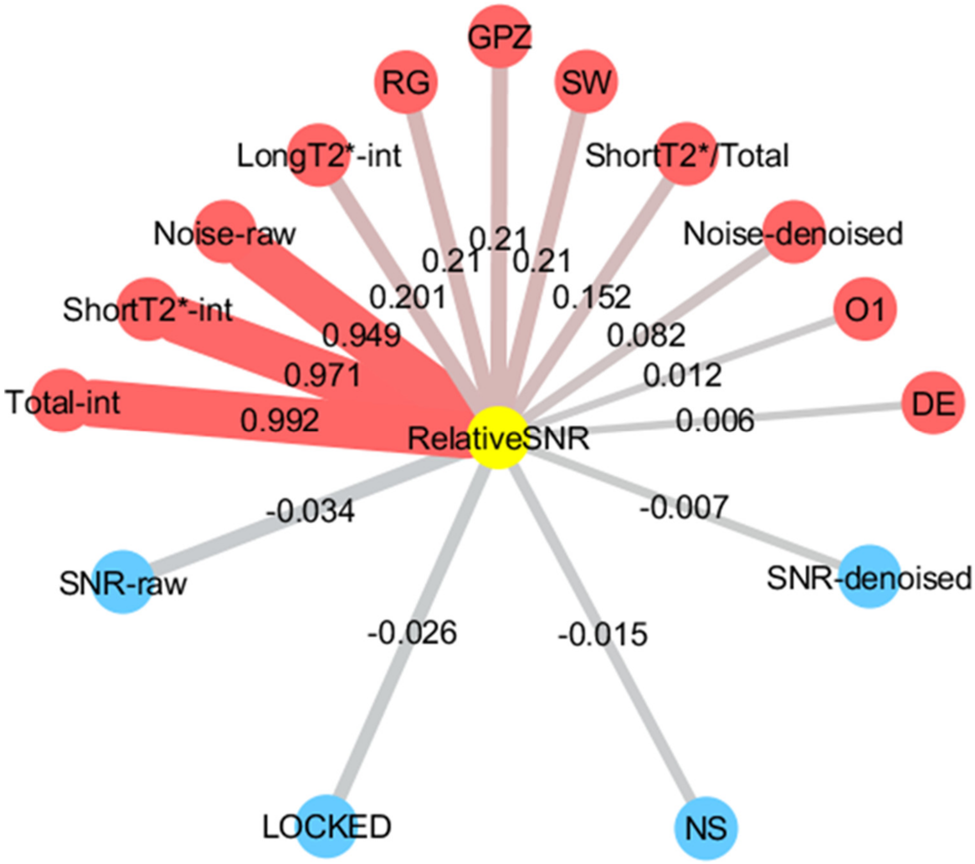

Figure S8b: Relationship between the data quality (SNR and the composition of the separated signal) and acquisition parameters of diffusion-edited NMR. b) Network diagram. In the network diagram, positive correlations are red; negative correlations are blue; and the magnitude of the correlation coefficient is indicated by edge thickness. Abbreviations: SNR-raw, SNR of raw data; SNR-denoised, SNR of denoised data; RelativeSNR, relative SNR; Total-int, total intensity; ShortT2*-int, intensity of short T2* signal; LongT2*-int, intensity of long T2* signal; ShortT2*/Total, ratio of intensity of long T2* signal to total intensity; Noise-raw, noise of raw data; Noise-denoised, noise of denoised data; GPZ, gradient pulse in the z-axis; RG, receiver gain; NS, number of scans; DE, pre-scan delay; SW, spectral width; O1, the offset of the transmitter frequency ; LOCKED, if LOCK is on, value is 1, if not, value is 0.

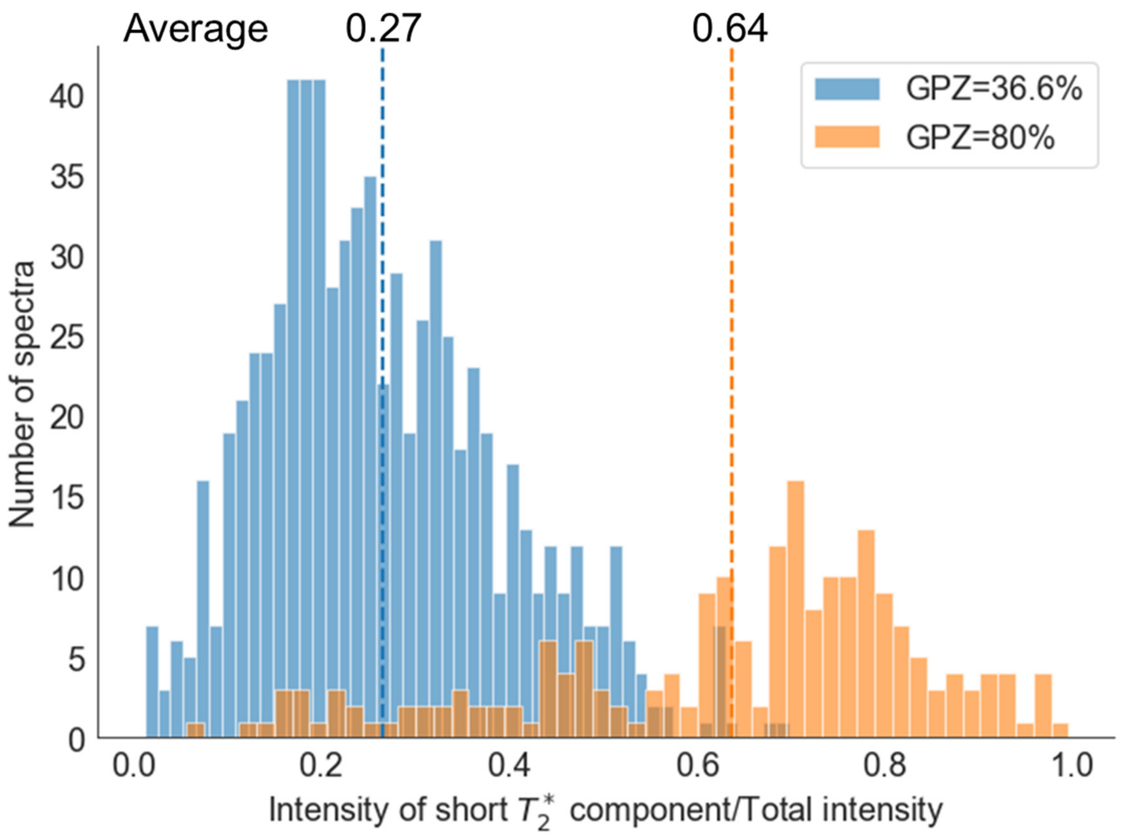

Figure S9: Histogram of the composition of the separated signal in diffusion-edited NMR data. We investigated the relationship between the composition of the separated signal and the gradient pulse in the *z*-axis (GPZ) parameter of diffusion-edited NMR. The histogram shows the relative SNR in NMR data measured using two different GPZ values. Shown is the ratio of the sum of short T_2 intensity to total intensity for GPZ = 36.6% (blue) and for GPZ = 80% (red). The average value in each pulse sequence is indicated.

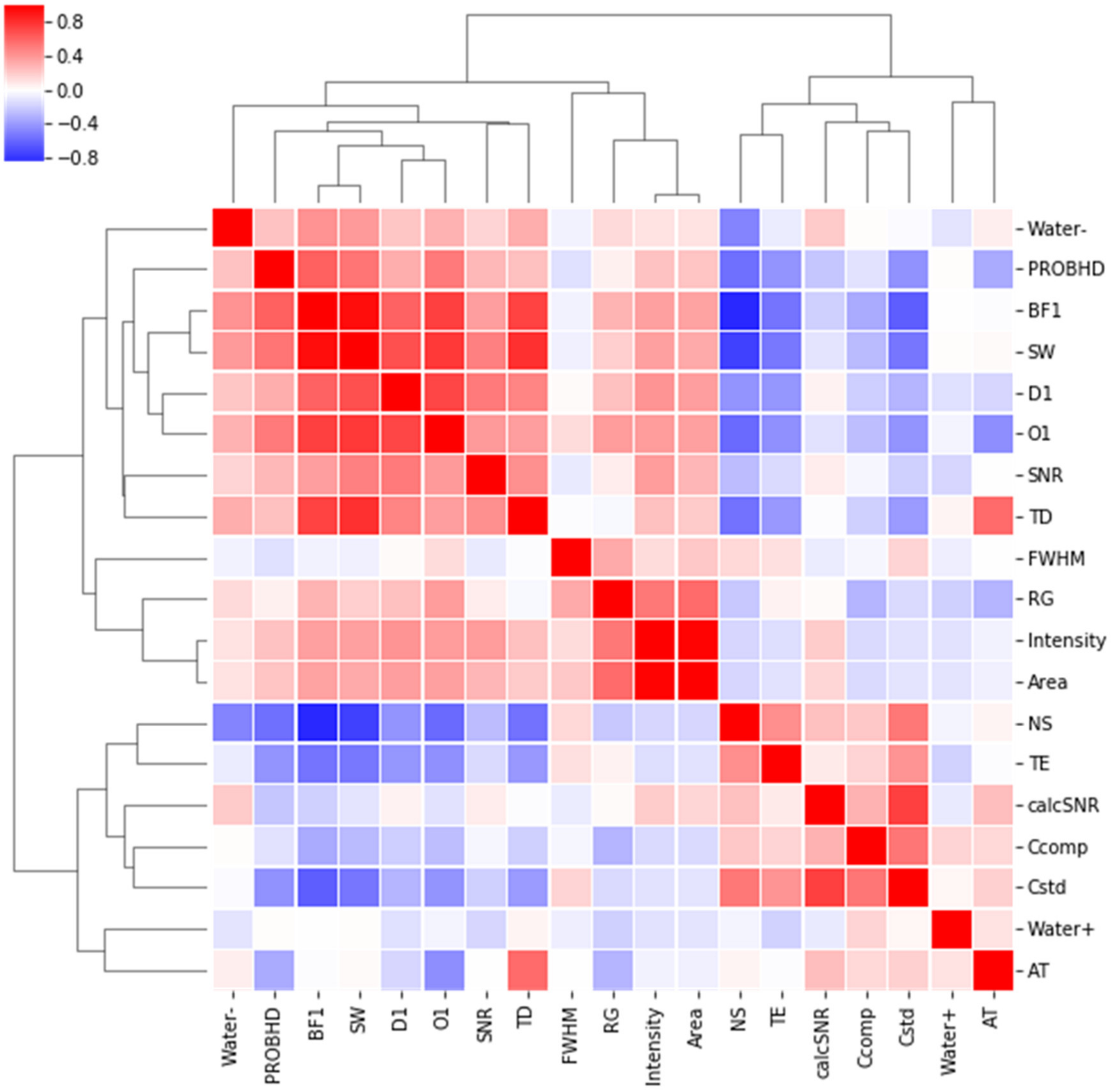

Figure S10: Heatmap summarizing correlation analysis between the data quality (SNR and signal values) and experimental parameters. Positive correlations are red; negative correlations are blue; and the magnitude of the correlation coefficient is shown as a color gradient. The parameters are clustered according to the similarity of their correlation coefficient as determined by hierarchical cluster analysis. Abbreviations: SNR, signal to noise ratio; calcSNR, calculated SNR; Cstd, concentration of standard compound; Ccomp, concentration of compound; Water+, positive intensity of water signal peak to standard peak; Water-, negative intensity of water signal peak to standard peak; Intensity, intensity of standard signal; FWHM, full width at half maximum; Area, area of standard signal; RG, receiver gain; NS, number of scans; D1, relaxation delay time; SW, spectral width; AT, acquisition time; TD, time-domain data size; O1, offset of transmitter frequency; TE, temperature; BF1, basic transmitter frequency for channel F1 in Hertz; PROBHD, if cryoprobe, value is 4, if not, value is 0.

Table S1: Original and denoised parameters and spectral values in citric acid data

^{1}H Chemical shift (ppm)		2.67	2.64	2.55	2.53	0
J value (Hz)		15.0		15.0		—
Original	Peak intensity	134991427	195407552	214161581	147849699	107410280
	FWHM (Hz)	2.40	2.48	2.31	2.26	2.13
Denoised	Peak intensity	134842313	194951941	213631581	147369942	107465227
	FWHM (Hz)	2.40	2.48	2.32	2.27	2.13
Error	Peak intensity (%)	0.11	0.23	0.25	0.32	-0.05
	FWHM (%)	0.02	-0.05	-0.52	-0.02	-0.04

^{1}H chemical shift, *J* value, peak intensity, and full width at half maximum (FWHM) are shown as the values of the original spectrum and the denoised spectrum in citric acid. Errors were calculated the difference between the original spectral value and the denoised spectral value. Relative SNR of this spectra is 1.14-fold.

Table S2: Summary of NMR spectra derived from sample ID of 1 to 10

Sample ID	PULPROG	D1	DE	NS	O1	RG	SW	TD	SNR-denoised	SNR-raw	Relative SNR	AT
1	CPMG	2	10	32	3457	108	14	32768	38229.09	14033.33	2.72	1.67
	Diffusion-edited	2	10	128	3291	388	16	16384	667.75	323.26	2.07	0.73
	Watergate	2.5	10	32	3295	108	14	32768	22718.07	5850.79	3.88	1.67
2	CPMG	2	10	32	3457	108	14	32768	1517567.61	504865.23	3.01	1.67
	Diffusion-edited	2	10	128	3291	388	16	16384	397.92	456.65	0.87	0.73
	Watergate	2.5	10	32	3295	108	14	32768	34829.85	8669.44	4.02	1.67
3	CPMG	2	10	32	3457	108	14	32768	1262994.59	443656.14	2.85	1.67
	Diffusion-edited	2	10	128	3291	388	16	16384	137.17	194.33	0.71	0.73
	Watergate	2.5	10	32	3295	108	14	32768	11642.91	4351.65	2.68	1.67
4	CPMG	2	10	32	3457	108	14	32768	102173.72	34671.49	2.95	1.67
	Diffusion-edited	2	10	128	3291	388	16	16384	679.61	246.47	2.76	0.73
	Watergate	2.5	10	32	3295	108	14	32768	15930.79	4331.21	3.68	1.67
5	CPMG	2	10	32	3457	108	14	32768	174450.86	77819.70	2.24	1.67
	Diffusion-edited	2	10	128	3291	388	16	16384	263.71	254.43	1.04	0.73
	Watergate	2.5	10	32	3295	108	14	32768	27185.68	6901.45	3.94	1.67
6	CPMG	2	10	32	3457	108	14	32768	155495.88	42460.54	3.66	1.67
	Diffusion-edited	2	10	128	3291	388	16	16384	617.51	306.23	2.02	0.73
	Watergate	2.5	10	32	3295	108	14	32768	15631.96	4608.96	3.39	1.67
7	CPMG	2	10	32	3457	108	14	32768	62782.12	29865.18	2.10	1.67
	Diffusion-edited	2	10	128	3291	388	16	16384	270.25	261.18	1.03	0.73
	Watergate	2.5	10	32	3295	108	14	32768	33748.08	7605.11	4.44	1.67

Sample ID	PULPROG	D1	DE	NS	O1	RG	SW	TD	SNR-denoised	SNR-raw	Relative SNR	AT
8	CPMG	2	10	32	3457	108	14	32768	100221.74	19528.38	5.13	1.67
	Diffusion-edited	2	10	128	3291	388	16	16384	1121.33	406.83	2.76	0.73
	Watergate	2.5	10	32	3295	108	14	32768	38506.44	7167.94	5.37	1.67
9	CPMG	2	10	32	3457	108	14	32768	54878.55	22587.59	2.43	1.67
	Diffusion-edited	2	10	128	3291	388	16	16384	1158.86	581.15	1.99	0.73
	Watergate	2.5	10	32	3295	108	14	32768	18295.45	7211.20	2.54	1.67
10	CPMG	2	10	32	3457	108	14	32768	58250.19	18693.05	3.12	1.67
	Diffusion-edited	2	10	128	3291	388	16	16384	271.59	265.08	1.02	0.73
	Watergate	2.5	10	32	3295	108	14	32768	32553.16	10245.24	3.18	1.67

Table S2 provides sample title, solvent and acquisition time, acquisition point, and original SNR as information about the sample and acquisition parameters. All data is available at http://dmar.riken.jp/NMRinformatics/SIforDCTN.zip. Abbreviations: PULPROG, pulse program used for the acquisition; D1, relaxation delay time; DE, pre-scan delay; NS, number of scans; O1, offset of transmitter frequency; RG, receiver gain; SW, spectral width; TD, time-domain data size; SNR-denoised, SNR of denoised data; SNR-raw, SNR of raw data; RelativeSNR, relative SNR; AT, acquisition time.

Table S3: FID datasets used for noise factor analysis

NMR	Bench-top NMR		High-field NMR								
	60 MHz		500 MHz				600 MHz			700 MHz	
Source	RIKEN	NUIS	RIKEN	BMRB	BML	HMDB	RIKEN	BMRB	HMDB	RIKEN	BMRB
Glucose	nanalysis (NMReady60PRO)	nanalysis (NMReady60PRO)	Bruker (c6-500c)	Bruker (MMC) [3]	Bruker (BML)	—	—	Bruker (MMC)	Varian (HMDB)	Bruker (c6-700b) [2]	Bruker (NIST)
Sucrose	nanalysis (NMReady60PRO)	nanalysis (NMReady60PRO)	Bruker (c6-500c)	Bruker (MMC) [2]	Bruker (BML) [2]	Varian (HMDB)	—	Bruker (MMC)	—	Bruker (c6-700b) [2]	Bruker (NIST)
Citric acid	nanalysis (NMReady60PRO)	nanalysis (NMReady60PRO)	Bruker (c6-500c)	Bruker (MMC) [3]	Bruker (BML)	Varian (HMDB)	—	—	—	Bruker (c6-700b) [2]	—
Lactic acid	nanalysis (NMReady60PRO)	nanalysis (NMReady60PRO)	Bruker (c6-500c)	Bruker (MMC) [3]	Bruker (BML)	Varian (HMDB) [2]	Bruker (c5-600c) [1]	Bruker (MMC)	—	Bruker (c6-700b)	Bruker (NIST)

We collected 48 sets of NMR data measured by low- and high-field NMR at multiple institutions to investigate the comprehensive relationship between noise and several acquisition parameters. Abbreviations: RIKEN, RIKEN Yokohama Campus; NUIS, Niigata University of International and Information Studies; BMRB, Biological Magnetic Resonance Data Bank; BML, Birmingham Metabolite Library; HMDB, Human Metabolome Database; MMC, Madison Metabolomics Consortium; NIST, National Institute of Standards and Technology. The NMR spectrometer manufacturer is listed; the product name, organization who generated the dataset, or control number is shown in parentheses. In the case of multiple data, the number of data used is indicated in square brackets.

3. References

1. Liu, H.; Dong, H.; Ge, J.; Liu, Z.; Yuan, Z.; Zhu, J.; Zhang, H. A fusion of principal component analysis and singular value decomposition based multivariate denoising algorithm for free induction decay transversal data. *Rev Sci Instrum* **2019**, *90*, 035116, doi:10.1063/1.5089582.
2. Keeler, J. Understanding NMR Spectroscopy. Appollo – University of Cambridge Repository: Cambridge, UK, 2004, doi:10.17863/CAM.1291.
3. Liu, H.; Dong, H.; Ge, J.; Bai, B.; Yuan, Z.; Zhao, Z. Research on a secondary tuning algorithm based on SVD & STFT for FID signal. *Meas Sci Technol* **2016**, *27*, 105006, doi:10.1088/0957-0233/27/10/105006.
4. Dueck, D.; Morris, Q.; Frey, B. Multi-way clustering of microarray data using probabilistic sparse matrix factorization. *Bioinformatics* **2005**, *21*, I144–I151, doi:10.1093/bioinformatics/bti1041.
5. Smith, J.O. *Mathematics of the Discrete Fourier Transform (DFT): with Audio Applications*, 2nd ed. BookSurge Publishing: South Carolina, USA, 2007.
6. Claridge, T. MNova: NMR data processing, analysis, and prediction software. *J Chem Inf Model* **2009**, *49*, 1136–1137, doi:10.1021/ci900090d.
7. Larive, C.K., Jayawickrama, D., Orfi, L. Quantitative analysis of peptides with NMR spectroscopy. *Appl Spectrosc* **1997**, 51, 1531–1536, doi:10.1366/0003702971939055.
